# A Rare Coexisting Presentation of Autosomal Dominant Polycystic Kidney Disease With Rapid Deterioration of Renal Function and Neurofibromatosis Type 1

**DOI:** 10.7759/cureus.79931

**Published:** 2025-03-02

**Authors:** Haruna Noishiki, Hiroko Yamauchi, Kazumi Komaki, Tetsuro Kusaba, Keiichi Tamagaki

**Affiliations:** 1 Nephrology, Graduate School of Medical Science, Kyoto Prefectural University of Medicine, Kyoto, JPN

**Keywords:** autosomal dominant polycystic kidney disease (adpkd), case report, chronic kidney disease (ckd), mtor system, neurofibromatosis 1 (nf1)

## Abstract

Autosomal dominant polycystic kidney disease (ADPKD) is one of the most common hereditary kidney disorders, characterized by progressive cyst development. Neurofibromatosis type 1 (NF1) is another autosomal dominant disorder, characterized by café-au-lait spots, neurofibromas, and multisystem involvement. We report the case of an 18-year-old male with ADPKD and NF1, referred due to progressively worsening renal dysfunction. His initial estimated glomerular filtration rate (eGFR) was 71.9 mL/min/1.73m², with MRI showing bilateral cystic renal enlargement (total kidney volume: 758 mL). One year later, his eGFR declined to 56.7 mL/min/1.73m², and kidney volume increased by 10.4% over one year. Tolvaptan was initiated, and he remains under follow-up.

Mutations in the PKD1/PKD2, which are responsible for ADPKD, affect intracellular signaling, including the mammalian target of the rapamycin (mTOR) pathway, leading to cyst formation and progression, while NF1 mutations overactivate the Ras proteins. His disease progression was more severe than that of his father with ADPKD alone, suggesting NF1 may have accelerated cyst enlargement. The co-occurrence of ADPKD and NF1 is extremely rare, with only a few cases reported in the past.

## Introduction

Autosomal dominant polycystic kidney disease (ADPKD) refers to the progressive development and enlargement of numerous cysts in the bilateral kidneys; it is the most frequent hereditary cystic kidney disease, with a prevalence of 1/2700 to 1/4000 [1]. About half of patients develop end-stage renal failure by the age of 60 years [1]. Abnormalities in polycystin 1 and 2 (PC1 and 2), encoded by PKD1 and PKD2, the causative genes of ADPKD, activate various signaling pathways, including the mammalian target of rapamycin (mTOR) system, leading to abnormal cell proliferation [1,2]. On the other hand, neurofibromatosis type 1 (NF1) is an autosomal dominant inherited disease that causes multiple lesions on the skin, bones, eyes, and nerves. The NF1 gene encodes a neurofibromin protein, which regulates Ras, one of the cancer-related genes [3]. Abnormal neurofibromin activates Ras excessively and promotes cell proliferation, leading to tumor formation and central nervous system symptoms. Since the frequency of this disease is rare (1 in 3,000) [4], only a few concurrent cases of NF1 and ADPKD have been reported [5-8]. We describe a case of NF1 combined with ADPKD, where the progression of ADPKD was more rapid than that of the patient's father who had ADPKD alone, suggesting that both conditions may have contributed to the deterioration of the disease.

## Case presentation

**Figure 1:**
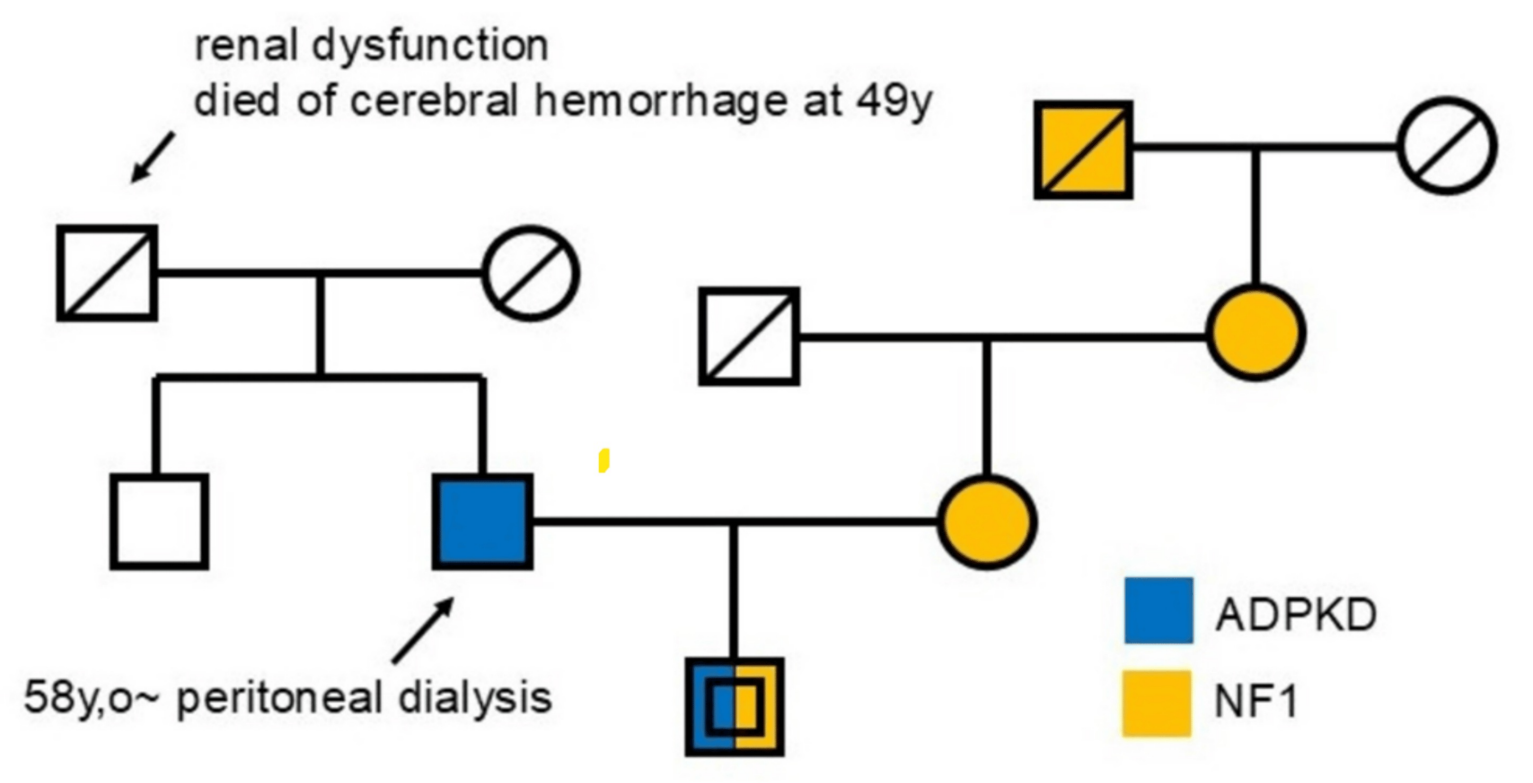
Family history of the patient NF1 was present on the maternal side of the patient's family, and ADPKD on the paternal side ADPKD: autosomal dominant polycystic kidney disease; NF1: neurofibromatosis type 1

An 18-year-old male was referred to nephrology from pediatrics due to progressive renal dysfunction. Despite annual follow-ups, the patient's serum creatinine (Cr) had gradually increased and exceeded 1.0 mg/dL a year ago, with his systolic BP rising above 130 since then. He had been diagnosed with NF1 in early childhood after developing café-au-lait spots and neurofibromas. At age two, he had been diagnosed with juvenile chronic myelogenous leukemia; he had been treated with a hematopoietic stem cell transplant (HSCT) at age three and had remained in remission. His mother, maternal grandmother, and maternal great-grandfather had NF1, while his father had ADPKD. His paternal grandfather had suffered from renal dysfunction and died of a cerebral hemorrhage at the age of 49 (Figure 1).

At the initial visit, his height was 161.8 cm; he weighed 51.3 kg, had a blood pressure of 138/96 mmHg, and a pulse of 82 beats per minute. His skin was covered with café-au-lait spots and some raised lesions. No abdominal distension or leg edema was observed. Blood tests suggested mild renal dysfunction [blood urea nitrogen (BUN): 19.8 mg/dL, Cr: 1.16 mg/dL, estimated glomerular filtration rate (eGFR): 71.9 mL/min/1.73m²], and urinalysis showed no abnormal findings. Abdominal ultrasound and MRI revealed numerous diffusely distributed bilateral renal cysts, with an estimated total kidney volume of 758 mL. No obvious renal artery stenosis was detected. Based on family history and imaging findings, ADPKD was diagnosed.

**Figure 2:**
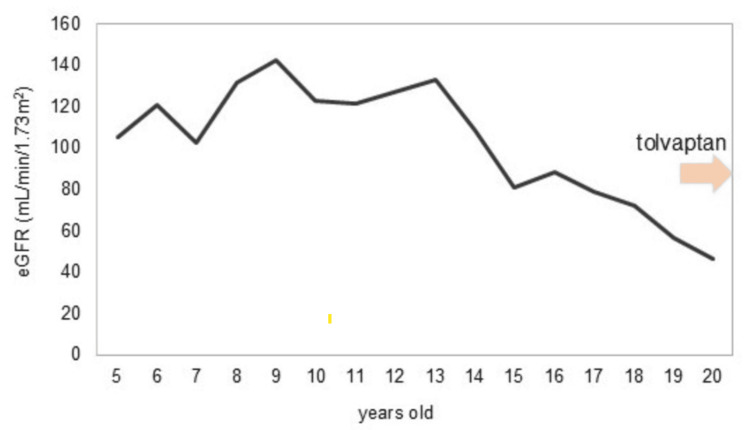
Course of renal function His renal function had gradually declined from around the age of 12

According to the Mayo classification [9], he was classified as Class 1E, reflecting the most severe disease progression. Head MRI showed no cerebral aneurysm, and echocardiography showed no valvular disease. Past blood test findings showed that eGFR had been declining since around age 12 (Figure 2).

Although the decrease remained within the normal range, no specific intervention for renal function was made. Hypertension was noted at the first visit, and antihypertensive treatment with amlodipine and olmesartan was initiated.

**Figure 3:**
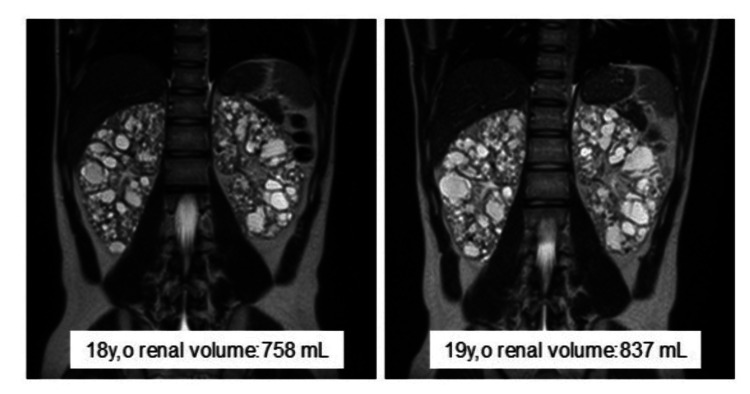
Abdominal MRI of the patient His kidney volume had increased from 758 mL to 857 mL over the course of a year MRI: magnetic resonance imaging

One year after the initial visit, his renal function declined significantly, with eGFR dropping from 71.9 to 56.7 mL/min/1.73m², and kidney volume increasing by 10.4% from 758 to 837 mL (Figure 3).

Treatment with tolvaptan was initiated at age 19, but renal function has continued to decline progressively. He undergoes bi-monthly follow-ups for renal function and blood pressure management. If renal function continues to decline, we are considering a living-donor kidney transplant with his mother as the donor.

Regarding the clinical course of his father, mild renal dysfunction (Cr: 1.16 mg/dL) had been first detected at age 46, and he had been diagnosed with ADPKD at 51. He took tolvaptan for a period of time at 52, but renal dysfunction had progressed slowly. He was initiated on peritoneal dialysis at age 58.

## Discussion

We discussed the case of a male patient with ADPKD combined with NF1. Compared to his father with ADPKD alone, this patient exhibited an earlier onset of renal dysfunction, rapid progression, and cystic enlargement. ADPKD is caused by PKD1 (85%) and PKD2 (15%) mutations, encoding PC1 and PC2 [1]. These proteins, localized in tubular epithelial cilia, regulate tubular diameter. Signals are transmitted from PC1, which acts as a sensor for urinary flow, to PC2, which acts as a Ca ion channel, causing Ca2+ to flow into the cell. When intracellular Ca2+ concentration is decreased due to abnormal PC1 and PC2, cAMP concentration in the tubular cells increases, and protein kinase A activity is elevated [10]. As a result, various signaling pathways including the mTOR system are activated, causing abnormal cell proliferation and increased secretion of cystic fluid, leading to cystic enlargement and eventually renal function deterioration [2].

The severity of ADPKD is primarily determined by the causative mutation. PKD1-truncating mutations (38%) are the most common and have the worst renal prognosis, followed by PKD2, PKD1 nontruncating, and PKD1 in-frame insertion/deletion mutations. PKD1 mutations are more severe than PKD2 mutations, and, in particular, PKD1-truncating mutations have the worst renal prognosis [11]. Even within families with the same genetic mutation, disease severity varies, indicating additional influencing factors. While environmental and lifestyle factors (e.g., diet, water intake, smoking, exercise) and comorbidities may play a role [12], a study on identical twins has found no difference in severity, suggesting a stronger impact of genetic modifiers [13]. These include transcriptional and translational regulators of PKD1/PKD2, modifier genes (e.g., cyst formation, type IV collagen), and genetic variants linked to chronic kidney disease (CKD) risk factors such as obesity, dyslipidemia, hypertension, and diabetes [11,12].

NF1, the coexisting disease in this case, is caused by mutations in the NF1 gene on chromosome 17, which encodes the neurofibromin protein. It is characterized by café-au-lait spots, neurofibromas, and multisystem involvement (skin, bones, eyes, nerves) with age. According to NIH diagnostic criteria, NF1 is confirmed if one criterion is met in individuals with a family history or two in those without. The criteria include neurofibromas, ≥6 café-au-lait macules (CALMs), optic glioma, Lisch nodules, axillary/inguinal freckling, distinctive bony lesions, or a pathogenic NF1 mutation [3]. Although genetic testing was not performed, this case fulfilled the NF1 diagnostic criteria based on family history and the presence of neurofibromas and CALMs.

Neurofibromin regulates Ras, one of the cancer-related genes [14]. In NF1, neurofibromin abnormalities result in the overactivation of Ras and activation of the Ras/MAP kinase pathway and the mTOR system. As a result, cell proliferation is promoted via the activation of the Ras/MAP kinase pathway and mTOR system, resulting in tumorigenesis and CNS symptoms [4]. We found that renal dysfunction and cyst enlargement occurred earlier in patients with both ADPKD and NF1 than in fathers with ADPKD alone. In a previous natural history study of ADPKD in children, the average renal volume at age 20 was about 300 mL [15], indicating that the renal volume in this case was notably large. As neurofibromin is expressed in tubules [16] and both neurofibromin and PC1 regulate Ras signaling [17], we speculate that NF1 may enhance ADPKD-driven mTOR signaling, potentially accelerating cyst enlargement.

The combination of ADPKD and NF1 is extremely rare, with an estimated incidence rate of 1 in 8.1-12 million based on the prevalence of both diseases, with only a few reported cases [5-8]. Peces et al. described a case with a PKD2 mutation, where no additive or synergistic effect on ADPKD progression was observed compared to a brother with ADPKD alone [8]. This may be due to PC2 dysfunction increasing cAMP and overactivating mTOR/S6K, while neurofibromin counteracts cAMP, preventing excessive cell proliferation.

In our case, genetic testing was not performed as ADPKD and NF1 can be definitively diagnosed based on family history, clinical symptoms, and imaging, and patient consent was not obtained. Given the father’s dialysis at age 58, PKD1 is highly likely. PC1 has been reported to directly suppress mTORC1 [18], potentially contributing to greater mTOR system hyperactivity compared to PKD2 and NF1 cases. As with ADPKD, differences in phenotype and severity are observed in NF1, with the main cause being the type of genetic mutation [19]. Peces et al.'s case had optic glioma but no skin neurofibroma, whereas our patient primarily had skin neurofibroma. A different NF1 mutation is expected, which may have influenced ADPKD progression differently.

Our patient had undergone HSCT for juvenile chronic myelogenous leukemia at age three. A follow-up study has reported chronic kidney disease in 17% of pediatric HSCT recipients after 10 years [20], and hence its involvement cannot be ruled out. However, the rapid cyst growth suggests NF1 may have contributed to mTOR activation by ADPKD. Tolvaptan suppresses cyst growth by inhibiting arginine vasopressin (AVP)-mediated cAMP production. However, in this case, mTOR and other cAMP-independent pathways may have contributed to cyst enlargement, limiting its effect. We are currently monitoring the patient's renal function and blood pressure bimonthly and performing annual renal MRIs, but the cysts continue to grow, and kidney function has declined. Gaining a better understanding of ADPKD progression mechanisms could help expand treatment options, emphasizing the need to accumulate more cases of ADPKD with NF1.

## Conclusions

We described a case of ADPKD and NF1 with early-onset cyst growth and progressive renal dysfunction. The variation in disease severity among family members, particularly the more rapid progression in our patient compared to his father with ADPKD alone, suggests that NF1 may have influenced cystic disease progression. Given that NF1 mutations are known to affect intracellular signaling pathways, including the mTOR pathway, their potential role in modulating ADPKD severity warrants further consideration. While additional studies are needed to clarify the impact of NF1 on ADPKD progression, accumulating similar cases may help improve our understanding of disease variability and guide future research on potential modifiers of ADPKD severity.

## References

[1] Bergmann C, Guay-Woodford LM, Harris PC, Horie S, Peters DJ, Torres VE (2018). Polycystic kidney disease. Nat Rev Dis Primers.

[2] Cornec-Le Gall E, Alam A, Perrone RD (2019). Autosomal dominant polycystic kidney disease. Lancet.

[3] Saleh M, Dib A, Beaini S (2023). Neurofibromatosis type 1 system-based manifestations and treatments: a review. Neurol Sci.

[4] Tamura R (2021). Current understanding of neurofibromatosis type 1, 2, and schwannomatosis. Int J Mol Sci.

[5] Chen MH, Chen KS, Hou JW, Lee CC, Huang JS (2002). Coexistence of autosomal dominant polycystic kidney disease and neurofibromatosis: report of a family. Am J Nephrol.

[6] Flego V, Radojcić Badovinac A, Plese V, Kapović M, Beg-Zec Z, Zaputović L (2003). Malignancy risk in patient with neurofibromatosis and autosomal dominant polycystic kidney disease. Croat Med J.

[7] Niemczyk S, Niemczyk M, Dylewska M, Gołębiowski M, Niemczyk L, Gomółka M (2010). Multiple intercostal neurofibromas in a patient with autosomal dominant polycystic kidney disease. Nephrology (Carlton).

[8] Peces R, Mena R, Martín Y (2020). Co-occurrence of neurofibromatosis type 1 and optic nerve gliomas with autosomal dominant polycystic kidney disease type 2. Mol Genet Genomic Med.

[9] Irazabal MV, Rangel LJ, Bergstralh EJ (2015). Imaging classification of autosomal dominant polycystic kidney disease: a simple model for selecting patients for clinical trials. J Am Soc Nephrol.

[10] Torres VE, Harris PC (2014). Strategies targeting cAMP signaling in the treatment of polycystic kidney disease. J Am Soc Nephrol.

[11] Hwang YH, Conklin J, Chan W (2016). Refining genotype-phenotype correlation in autosomal dominant polycystic kidney disease. J Am Soc Nephrol.

[12] Yeung KC, Fryml E, Lanktree MB (2024). How does ADPKD severity differ between family members?. Kidney Int Rep.

[13] Persu A, Duyme M, Pirson Y (2004). Comparison between siblings and twins supports a role for modifier genes in ADPKD. Kidney Int.

[14] Takano T, Kawashima T, Yamanouchi Y, Kitayama K, Baba T, Ueno K, Hamaguchi H (1992). Genetics of neurofibromatosis 1 in Japan: mutation rate and paternal age effect. Hum Genet.

[15] Fick-Brosnahan GM, Tran ZV, Johnson AM, Strain JD, Gabow PA (2001). Progression of autosomal-dominant polycystic kidney disease in children. Kidney Int.

[16] (2025). The Human Protein Atlas. https://www.proteinatlas.org/.

[17] Rosner M, Hanneder M, Siegel N, Valli A, Fuchs C, Hengstschläger M (2008). The mTOR pathway and its role in human genetic diseases. Mutat Res.

[18] Reiterová J, Tesař V (2022). Autosomal dominant polycystic kidney disease: from pathophysiology of cystogenesis to advances in the treatment. Int J Mol Sci.

[19] Borofsky S, Levy LM (2013). Neurofibromatosis: types 1 and 2. AJNR Am J Neuroradiol.

[20] Lugthart G, Jordans CC, de Pagter AP (2021). Chronic kidney disease ten years after pediatric allogeneic hematopoietic stem cell transplantation. Kidney Int.

